# *Streptococcus pneumoniae* Carriage in the Gaza Strip

**DOI:** 10.1371/journal.pone.0035061

**Published:** 2012-04-23

**Authors:** Gili Regev-Yochay, Izzeldin Abullaish, Richard Malley, Bracha Shainberg, Miriam Varon, Yulia Roytman, Arnona Ziv, Aviva Goral, Abedallah Elhamdany, Galia Rahav, Meir Raz, for the Palestinian-Israeli Collaborative Research (PICR) study group

**Affiliations:** 1 Infectious Disease Unit, Sheba Medical Center, Tel-Hashomer affiliated to the Sackler School of Medicine, Tel-Aviv University, Tel-Aviv, Israel; 2 Gertner Institute for Epidemiology Research, Tel-Hashomer, Ramat-Gan, Israel; 3 Division of Infectious Diseases, Children's Hospital Boston and Harvard Medical School, Boston, Massachusetts, United States of America; 4 Central Laboratory, Macabbi Healthcare Services, Rehovot, Israel; 5 Jerusalem-Hashfela District, Macabbi Healthcare Services, Modiin, Israel; 6 Toronto University, Dalla Lana School of Public Health, Toronto, British Columbia, Canada; 7 Keystone Consulting Agency, Gaza City, the Gaza Strip, Palestinian Territories; Instituto Butantan, Brazil

## Abstract

**Background:**

Pneumococcal infections cause major morbidity and mortality in developing countries. We report the epidemiology of *S. pneumoniae* carriage in a developing region, the Gaza strip, and evaluate the theoretical coverage of carriage strains by pneumococcal conjugate vaccines (PCVs).

**Methodology:**

In 2009 we conducted a cross-sectional survey of *S. pneumoniae* carriage in healthy children and their parents, living throughout the Gaza strip. Data were collected and nasopharyngeal swabs were obtained. Antibiotic susceptibilities were determined by Vitek-2 and serotypes by the Quellung reaction.

**Principal Findings:**

*S. pneumoniae* carriage was detected in 189/379 (50%) of children and 30/376 (8%) of parents. Carriage prevalence was highest in children <6 months of age (63%). Significant predictors for child carriage were number of household members and DCC attendance. The proportion of pediatric and adults isolates with serotypes included in PCV7 were 32% and 20% respectively, and 46% and 33% in PCV13 respectively. The most prominent non-vaccine serotypes (NVT) were 35B, 15B/C and 23B. Penicillin-nonsusceptible strains were carried by70% of carriers, penicillin-resistant strains (PRSP) by 13% and Multi-drug-resistant (MDR) by 30%. Of all PRSP isolates 54% belonged to serotypes included in PCV7 and 71% in the PCV13. Similarly, 59% and 73% of MDR-SP isolates, would theoretically be covered by PCV7 and PCV13, respectively.

**Conclusions:**

This study demonstrates that, PCV13-included strains were carried by 46% and 33% of pediatric and adult subjects respectively. In the absence of definitive data regarding the virulence of the NVT strains, it is difficult to predict the effect of PCVs on IPD in this region.

## Introduction

*S. pneumoniae* is the primary cause of bacterial infections in children, causing over 860,000 deaths a year in children worldwide [1]. Nasopharyngeal colonization is the first and necessary stage prior to infection, and serves as the source of transmission between person to person [2]. Thus, to understand the epidemiology and spread of pneumococcal infections it is important to study the epidemiology of *S. pneumoniae* colonization.

Since 2000, universal vaccination with the 7-valent pneumococcal conjugate vaccine (PCV7) of children <5 years has been implemented in the USA. Following the introduction of this vaccine, a dramatic decrease in invasive pneumococcal infections was observed in the USA [3]. Since most developing countries have not yet implemented universal vaccination with this vaccine, it is as yet unknown whether a similar effect will be observed in countries where the incidence of disease is even higher.

The Palestinian-Israeli collaborative research (PICR) group was established to study the direct, indirect and potential effects of PCV7 on Israeli and Palestinian populations.

The Gaza strip is a 360-km^2^ narrow area located on the Eastern coast of the Mediterranean Sea. The strip borders Egypt on the south, the Mediterranean on the west and Israel on the north and east. It is populated by about 1.5 million inhabitants. The annual population growth rate is 3.3%, and the infant mortality rate is ∼20/1000 (UNRWA data: http://unispal.un.org/UNISPAL.NSF/0/885BD85F892778F28525772700503A4B). Poverty, large families (average family size, 7.5persons) and overcrowding are prevalent. Health care is provided by the Palestinian Ministry of Health Services, and the United Nations Relief and Works Agency (UNRWA) Health Department as well as private and charitable sources. No PCV has yet been introduced in the Gaza strip; the implementation of universal vaccination with either PCV7, PCV10 or PCV13 in this region is currently under consideration by the Palestinian Authority.

The epidemiology of *S. pneumoniae* carriage in the Gaza strip has not yet been studied. In this study we assessed the prevalence of *S. pneumoniae* carriage in the Gaza strip.

## Methods

### Study population and site

The surveillance took place between March and July 2009.Children under 5.5 years of age, who lived in 12 neighborhoods or villages in north and central Gaza strip were randomly selected. The parents were contacted and asked to participate in the study; if consent was given, a research assistant then visited the family to obtain signed consent. Only one child and one parent were enrolled from each family. The study was approved by the institutional review boards of Macabbi Healthcare Services and the Gaza ministry of health.

### Data Collection

Parents were interviewed to collect the following demographic and medical information: age and gender of the child and parent, number of household members, number of siblings, age of siblings, day care attendance of child or his siblings, working place of the parent, smoking by any of the household members, breastfeeding, owning pets, medical history of the child and the parent including any chronic or recurrent illnesses, antibiotic use during the previous month, immunizations, hospitalization during the past 6 months and medical status on the screening day.

### *S. pneumoniae* screening

A single nasopharyngeal swab, using a rayon-tipped aluminum shaft swab placed in Amies transport media (Copan innovation, Brescia, Italy) was collected. Within 24 h, specimens were transported to the Macabbi Healthcare Services central laboratory in Israel and streaked onto tryptic soy agar plates supplemented with sheep-blood and 5 µg/ml gentamicin and were incubated overnight at 35°C in 5% CO_2_enriched air. *S. pneumoniae* was identified using morphological characteristics, α-hemolysis and optochin susceptibility. Antibiotic susceptibility was determined by Vitek 2. A strain was defined as penicillin non-susceptible if the minimum inhibitory concentration (MIC) to penicillin was >0.06 mg/L and penicillin resistance was defined as MIC≥2 mg/L. Multi-drug resistance was defined as non-susceptibility to at least penicillin (MIC>0.06 mg/L) and erythromycin (MIC>0.5 mg/L). Serogroup was determined by the latex agglutination test and the serotype was determined by the Quellung reaction (Staten Serum Institute, Copenhagen, Denmark).

### Statistical Analyses

Analyses were performed using SAS software package, version 9.2 (SAS Institute). Frequencies of potential predictors were compared using χ^2^ or Fisher's exact test when appropriate. Median values were compared using the Mann-Whitney U test. Results were considered statistically significant if the 2-tailed P value was <0.05. Variables assessed as predictors for carriage, or for carriage of resistant strains included; age, gender, number of household members, day care center (DCC) attendance, antibiotic use during the month prior to screening, and hospitalization during 6 months prior to screening. In the multivariate logistic model, variables that were significant or trended towards significance (p<0.2) were included and the adjusted odds ratio (aOR) was assessed.

## Results

### Patient characteristics

Overall, 758 individuals were enrolled: 379 children and one of their parents. Children's age ranged from 3 weeks to 5.5 years with a median age of 1.86 years; nearly one quarter of the children (24%) were younger than 1 year. Female/male ratio was about 1;1. Per parental report, almost all children (96%) stayed at home with their siblings and their family and did not attend DCC. The median number of household members was 7 with the range of 2–25. Fewer than 10% of the children (26, 6.9%) had no siblings while a quarter (97 children, 25.6%) of the children had six or more siblings. Most children (95%) completed the routine immunization schedule in the Gaza strip (i.e. Hepatitis B, BCG, OPV + IPV, DPT, Hib, MMR). No child was immunized with PCV7, which, at the time of the study, was not available in the Gaza strip. The study parents' age ranged from 19.5 to 58 years with a median of 32 years. Most of the screened parents (69%) were mothers of which 94% did not work outside of the home. Among the 106 fathers screened 37% reported that they were unemployed.

### *S. pneumoniae* carriage

**Figure 1:**
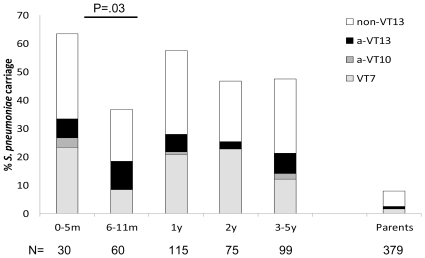
*S. pneumoniae* carriage by age and by PCV7 vaccine type (VT7) serotypes, serotypes added to PCV7 in PCV10 (a-VT10), serotypes added to PCV10 in PCV13 (a-VT13). Light grey-VT7 serotype carriage. Dark grey-a-VT10 (1, 5, 7F), Black – a-VT13 (3, 6A, 19A), White - Non-VT13 carriage.

*S. pneumoniae* carriage was detected in 50% (189/379) of children and 8% (30/376) of parents. Carriage prevalence was highest in children <6 months with 63% (19/30) carriage and lowest in children aged 6–11 m with 37% (22/60) carriage. In older age groups the carriage prevalence varied between 47% and 57% (**Figure 1**).Predictors of *S. pneumoniae* carriage included number of household members (aOR for each additional member 1.08; 95% confidence interval (CI) 1.01–1.14, p = 0.02) and DCC attendance (aOR 3.72; 95%CI 0.98–14.17, p = 0.05), while age 6–11 m was a negative predictor (aOR 0.36; 95%CI 0.14–0.92, p = 0.003)(**Table 1**).

**Table 1:** Predictors of *S. pneumoniae* carriage in children in the Gaza strip.

Predictor		N*	*% S. pneumoniae*	aOR	p-
			carriage**	(95%CI)	value
Age	<6months	30	63.33%	REF	
	6–11 m	60	36.7%	.36	.003
				(.14–.92)	
	1 y	115	56.5%	.81	.63
				(.35–1.90)	
	2 y	75	46.7%	0.53	.16
				(.22–1.28)	
	3–5.5 y	99	47.5%	.47	.09
				(.20–1.12)	
Gender	Male	187	49.2%		
	Female	192	49.5%		
*S.pneumoniae*	No	345	48.7%	REF	
carriage by parent	Yes	30	60.0%	1.31	.51
				(.59–2.94)	
Attending DCC	No	366	48.6%	REF	
	Yes	13	76.9%	3.72	.05
				(.98–14.17)	
Received Abx in	No	237	51.1%	REF	
past 6 months	Yes	142	47.2%	0.86	.49
				(.55–1.33)	
Hospitalization in	No	327	48.6%		
previous 6 months	Yes	52	55.8%		
Number of	each extra			1.08	.02
household members	member			(1.01–1.14)	
Smoker	No	257	48.1%		
	Yes	116	54.2%		
Current	No	95	50.5%		
Breastfeeding	Yes	103	54.4%		
(amongchildren<2 y)					

* N = total number of children with the specified predictor.

** % *S. pneumoniae* carriage among children with the specific predictor.

### Potential coverage by PCV7

**Figure 2:**
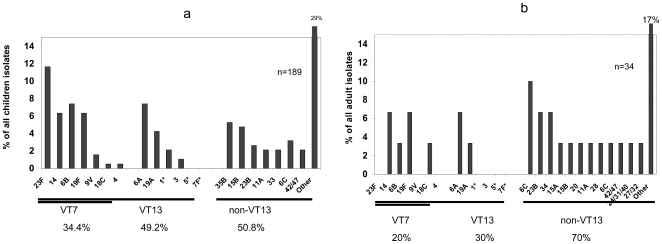
Serotype distribution among carriage strains in the Gaza strip. a. Among children. b. Among parents. Vaccine type (VT)7: serotypes covered by PCV7, VT13: serotypes covered by PCV13. Asterix (*) - serotypes covered by PCV10 (1, 5, 7F), Non-VT13: only serotypes that are prevalent in >2% are depicted separately.

Only 34.4% of carried *S. pneumoniae* isolates in children belonged to serotypes included in PCV7 (referred to as vaccine-types (VT7)), while among parents the proportion of VT7 isolates was even lower (20.0%). While 36.5% of children's' isolates and 20.0% of parents' isolates were VT10, 49.2% and 30.0% of children's' and parents' isolates respectively were VT13 strains (**Figure 2**). The most frequent non-VT13 serotypes in children were 35B (5.3% of all strains), 15B (4.8%) and 23B (2.7%), while in adults a large diversity of non-VT13 serotypes was observed, the most common being 6C (10.0% of all adult strains).

### Antibiotic use and antibiotic resistance

Antibiotic use within the month prior to screening was reported in 37.5% of children and 17.4% of parents. Among the cases in which the antibiotic class that was used was known (45% of cases), the most frequently used in children was penicillin (64%), followed by cephalosporins (22%); macrolide use was relatively rare (6%).

**Figure 3:**
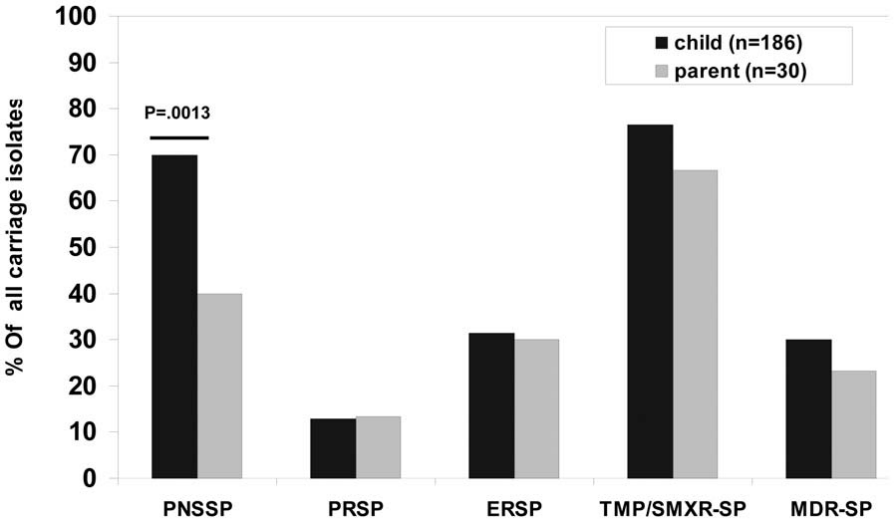
Prevalence of antibiotic resistance among isolates. Black - children. Grey - Parents. PNSSP: penicillin non-susceptible *S. pneumoniae* (MIC>0.06 mg/L), PRSP: penicillin resistant *S. pneumoniae* (MIC≥2.0 mg/L), ERSP: erythromycin resistant *S. pneumoniae* (MIC>0.5 mg/L), TMP/SXTR-SP: trimethoprim-sulfamethoxazolenon-susceptible *S. pneumoniae* (MIC>0.5 mg/L),MDR-SP: multi-drug resistant *S. pneumoniae*, defined as non-susceptible to at least penicillin and erythromycin.

Among pediatric carriage strains, 70.1% (132) were penicillin-non-susceptible (PNSSP) and 12.8% (24) were penicillin-resistant (PRSP). Multi-drug-resistant (MDR)*S. pneumoniae* strains were carried by 56 children; this represented 30.0% of pediatric carriage strains. The prevalence of PNSSP among carrier parents was lower than in children (40.0%vs. 70.1%, p = 0.0013), but PRSP and erythromycin-resistance prevalence were similar to those in children (**Figure 3**).

The only independent predictor for PRSP or MDR-SP carriage by children in a multivariate logistic analysis was recent antibiotic treatment (aOR = 3.72, 95%CI 1.48–9.35 (p = .005) and aOR = 3.27, 95%CI 1.62–6.60 (p = .001) respectively). For MDR carriage in children, male gender was also a predictor for carrying MDR-SP (aOR = 2.18, 95%CI 1.08–4.41, p = .003). No specific antibiotic class could be defined as a particular predictor for either antibiotic resistance patterns, but the small number of children in each individual category may have limited the power to detect any association.

### Potential effect of PCVs on reducing antibiotic resistance

**Figure 4:**
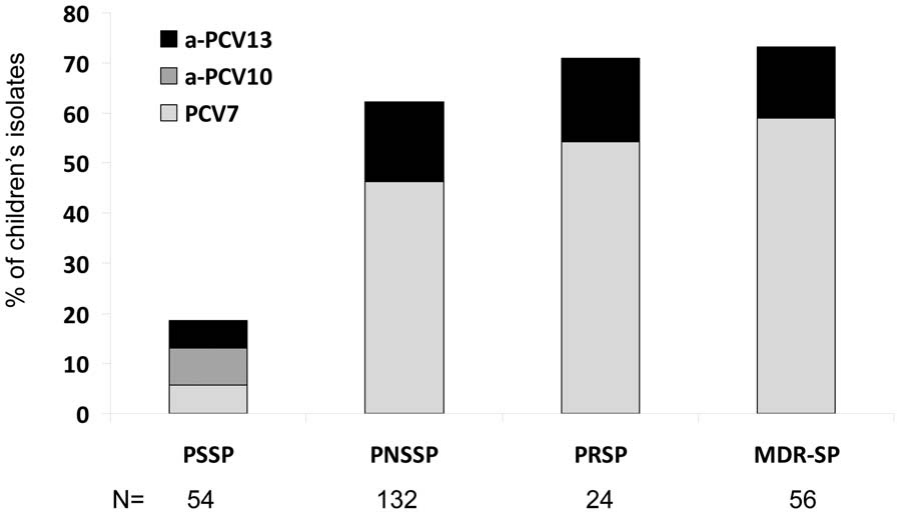
Potential coverage of antibiotic resistant strains in children by PCV7 (Light Grey), PCV10 (Light+Dark Grey) and PCV13 (Light+Dark Grey+Black). PSSP: penicillin susceptible *S. pneumoniae*, PNSSP: penicillin non-susceptible *S. pneumoniae* (MIC>0.06 mg/L), PRSP: penicillin resistant *S. pneumoniae* (MIC≥2.0 mg/L), MDR-SP: multi-drug resistant *S. pneumoniae*, defined as non-susceptible to at least penicillin and erythromycin.

To assess the potential effect of PCVs on reducing the frequency of antibiotic resistance in children, we examined the proportion of PRSP and MDR-SP that belong to VT7, VT10 or VT13 strains. Of all PRSP isolates 54.2% belonged to serotypes included in PCV7, or PCV10 and 70.9% in the PCV13. Similarly, 58.9%of MDR-SP isolates belong to VT7 or VT10 and 73.2% of MDR-SP belong toVT13 serotypes (**Figure 4**). Of the 56 MDR-SP isolates, the most common serotypes were 19F (17.9% of MDR isolates) followed by 14, 6B and non-typeable serotypes (16.1% each); 15 MDR-SP isolates (26.8%) had serotypes that would not be covered by PCV13 (non-VT13). The serogroup with the highest likelihood of antibiotic resistance was 14: 66.7% of serogroup 14 isolates were PRSP and 75.0% MDR; serotype 19F also had a high likelihood of resistance, with 33.3% PRSP and 83.3% MDR. Fifty percent of serotype 19A isolates were PRSP and 37.5% were MDR. PCV7 would cover most PNSSP except for serotypes 6A, 19A 35B and 15B; the first two serotypes would in theory be covered by PCV13. Assuming similar effects of PCV7 on colonizing strains [4], [5], [6], [7], [8], PCV7 and PCV10 could potentially decrease 54.2%, 58.9% and 46.2% of PRSP, MDR and PNSSP pediatric isolates, respectively; the theoretical impact of PCV13 would be a decrease of 70.9%, 73.2% and 62.1%, respectively.

## Discussion

In this study we report data on *S. pneumoniae* carriage in Palestinian populations living in the Gaza strip. We show that *S. pneumoniae* carriage is high, particularly at a very early age. Previous studies have showed that children below 3 months of age are rarely *S. pneumoniae* carriers and that the most significant risk factor for carriage is attending DCC [9], [10], [11]. The findings in the Gaza strip are different, in that very few children attend DCC, and yet *S. pneumoniae* carriage was detected at a very early age. These findings are overall consistent with those obtained from other developing countries, and have been attributed to the low socioeconomic and high overcrowding conditions [12], [13], [14]. An intriguing finding in our study is the significantly lower carriage prevalence among children 6–11 months old. This unexpected finding persists even after adjusting for potential effects of breastfeeding or DCC attendance, a result that remains to be explained.

The relatively low coverage of colonizing serotypes by PCV7 in the Gaza strip shown here mirrors findings from over a decade ago [15] and more recent studies of invasive disease in developing world settings. In that study 97 isolates from middle ear fluid (MEF) or CSF were assessed, 47% and 24% of isolates from MEF and CSF, respectively, were found to belong to PCV7 strains. A recent study of bacterial infections in children in Nigeria identified 21 cases of IPD, in which PCV7 would cover 55% of cases [16]. A study on bacterial meningitis in Uganda identified 43% of serotyped *S. pneumoniae* isolates to be covered by PCV7 [17] and a study that followed 99 children in Bangladesh [18], showed approximately 48% PCV7 coverage, but serotypes were not defined, and serogroups were used to approximate PCV7 coverage.

There has been a call for the rapid implementation of PCV7 in developing countries [19], mainly based on the success of this vaccine in the US and Europe, and two clinical trials that have demonstrated impressive efficacy of PCV9 in developing countries [20], [21]. How effects observed in controlled clinical trials will translate to situations of universal immunization in developing regions is unclear and will need to be studied [22]. Since January 2010 two countries supported by Gavi alliance for pneumococcal vaccines have introduced PCV7 into routine childhood immunization (the Gambia and Rwanda) [23]; the results of this important intervention are eagerly awaited.

We readily acknowledge that the limitation that our study focuses on carriage serotypes only and not invasive disease strains. However, carriage data has been used to estimate IPD using the relative invasive capacity of the different serotypes [24]. It appears that the invasive capacity of serotypes do not vary significantly across different populations [25], [26], [27], and thus carriage data could well be used to predict the potential effect of PCVs in this region as well.

On the other hand, the possibility remains that despite the relatively low coverage by PCV7 of pediatric carriage strains in the Gaza strip, the introduction of a PCV in the region could have an important clinical impact. While it appears that PCV7 will cover only 30% of pediatric colonization strains, a 30% decrease in these strains may translate to more reduction of disease, since it appears that these VT7 strains are associated with more virulence and morbidity than some of the other NVT strains [3], [22], [28]. An unknown factor, and one that cannot be readily ascertained from the result of vaccine trials, is to what extent and how fast will replacement disease by non-VT strains occur following universal immunization. It is also unclear whether the extent of replacement is dependent on the pre-vaccination PCV7 coverage [22] (30% in Gaza compared to ∼90% in USA).

It has also been reported that pneumococcal carriage that occurs shortly before the first PCV7 dose may result in hypo-responsiveness to the carried strain serotype [29], a finding which, if applicable broadly, may have a greater impact in developing countries and in the Gaza strip, where colonization occurs at a much earlier age.

Another potential benefit of the introduction of PCVs would be to reduce antibiotic resistance, as has been noted in several countries [6], [30], [31], [32], [33]. It should be noted however that, in regions where PCV7 has been implemented and antibiotic resistance patterns were followed, the effect of PCV7 on lowering antibiotic resistance tend to be rather short-lived, if not accompanied by concurrent reduction in antibiotic use [7], [34], [35], [36]. High antibiotic use was reported in our study population. Since only 45% of the subjects who reported antibiotic use could specify the antibiotic name or class used, we cannot be certain that there may have been some misclassification of other medicines as antibiotics by the subjects. That said, the high antibiotic resistance prevalence is indeed suggestive of actual high antibiotic consumption. In any case, a concerted effort to reduce antibiotic use should also be included in a vaccination campaign in the Gaza strip.As in other parts of the world, much of the antibiotics in this region are obtained by over-the-counter purchase, which may complicate any efforts to curtail inappropriate antibiotic use.

In conclusion, our study reports the prevalence of *S. pneumoniae* carriage in the Gaza strip, the prevalence of VT7, VT10 and VT13 strains and the prevalence of antibiotic resistant strains. As has been observed in other settings in the developing world, the potential coverage of PCVs against colonization strains is not as high as in the US or Western Europe. The evolving epidemiology of pneumococcal colonization and disease in the Gaza strip will need to be monitored closely and over time, to assess the real impact of the introduction of PCVs in this region.

## References

[1] O'Brien KL, Wolfson LJ, Watt JP, Henkle E, Deloria-Knoll M (2009). Burden of disease caused by Streptococcus pneumoniae in children younger than 5 years: global estimates.. Lancet.

[2] Bogaert D, De Groot R, Hermans PW (2004). Streptococcus pneumoniae colonisation: the key to pneumococcal disease.. Lancet Infect Dis.

[3] Pilishvili T, Lexau C, Farley MM, Hadler J, Harrison LH (2010). Sustained reductions in invasive pneumococcal disease in the era of conjugate vaccine.. J Infect Dis.

[4] Flasche S, Van Hoek AJ, Sheasby E, Waight P, Andrews N (2011). Effect of pneumococcal conjugate vaccination on serotype-specific carriage and invasive disease in England: a cross-sectional study.. PLoS Med.

[5] Spijkerman J, van Gils EJ, Veenhoven RH, Hak E, Yzerman EP (2011). Carriage of Streptococcus pneumoniae 3 years after start of vaccination program, the Netherlands.. Emerg Infect Dis.

[6] Vestrheim DF, Hoiby EA, Aaberge IS, Caugant DA (2010). Impact of a pneumococcal conjugate vaccination program on carriage among children in Norway.. Clin Vaccine Immunol.

[7] Huang SS, Hinrichsen VL, Stevenson AE, Rifas-Shiman SL, Kleinman K (2009). Continued impact of pneumococcal conjugate vaccine on carriage in young children.. Pediatrics.

[8] Huang SS, Platt R, Rifas-Shiman SL, Pelton SI, Goldmann D (2005). Post-PCV7 changes in colonizing pneumococcal serotypes in 16 Massachusetts communities, 2001 and 2004.. Pediatrics.

[9] Regev-Yochay G, Raz M, Shainberg B, Dagan R, Varon M (2003). Independent risk factors for carriage of penicillin-non-susceptible Streptococcus pneumoniae.. Scand J Infect Dis.

[10] Labout JA, Duijts L, Arends LR, Jaddoe VW, Hofman A (2008). Factors associated with pneumococcal carriage in healthy Dutch infants: the generation R study.. J Pediatr.

[11] Regev-Yochay G, Raz M, Dagan R, Porat N, Shainberg B (2004). Nasopharyngeal carriage of Streptococcus pneumoniae by adults and children in community and family settings.. Clin Infect Dis.

[12] Millar EV, O'Brien KL, Zell ER, Bronsdon MA, Reid R (2009). Nasopharyngeal carriage of Streptococcus pneumoniae in Navajo and White Mountain Apache children before the introduction of pneumococcal conjugate vaccine.. Pediatr Infect Dis J.

[13] Jacoby P, Carville KS, Hall G, Riley TV, Bowman J (2011). Crowding and other strong predictors of upper respiratory tract carriage of otitis media-related bacteria in Australian Aboriginal and non-Aboriginal children.. Pediatr Infect Dis J.

[14] Hill PC, Akisanya A, Sankareh K, Cheung YB, Saaka M (2006). Nasopharyngeal carriage of Streptococcus pneumoniae in Gambian villagers.. Clin Infect Dis.

[15] el-Astal Z, Khamis N, Peled N, Dagan R, Yagupsky P (1997). Antimicrobial resistance and typing of pneumococci in Gaza Strip children.. Pediatr Infect Dis J.

[16] Falade AG, Lagunju IA, Bakare RA, Odekanmi AA, Adegbola RA (2009). Invasive pneumococcal disease in children aged <5 years admitted to 3 urban hospitals in Ibadan, Nigeria.. Clin Infect Dis.

[17] Kisakye A, Makumbi I, Nansera D, Lewis R, Braka F (2009). Surveillance for Streptococcus pneumoniae meningitis in children aged <5 years: implications for immunization in Uganda.. Clin Infect Dis.

[18] Granat SM, Mia Z, Ollgren J, Herva E, Das M (2007). Longitudinal study on pneumococcal carriage during the first year of life in Bangladesh.. Pediatr Infect Dis J.

[19] Levine OS, O'Brien KL, Knoll M, Adegbola RA, Black S (2006). Pneumococcal vaccination in developing countries.. Lancet.

[20] Cutts FT, Zaman SM, Enwere G, Jaffar S, Levine OS (2005). Efficacy of nine-valent pneumococcal conjugate vaccine against pneumonia and invasive pneumococcal disease in The Gambia: randomised, double-blind, placebo-controlled trial.. Lancet.

[21] Klugman KP, Madhi SA, Huebner RE, Kohberger R, Mbelle N (2003). A trial of a 9-valent pneumococcal conjugate vaccine in children with and those without HIV infection.. N Engl J Med.

[22] Weinberger DM, Malley R, Lipsitch M (2011). Serotype replacement in disease after pneumococcal vaccination.. Lancet.

[23] Kim SY, Lee G, Goldie SJ (2010). Economic evaluation of pneumococcal conjugate vaccination in The Gambia.. BMC Infect Dis.

[24] Yildirim I, Hanage WP, Lipsitch M, Shea KM, Stevenson A (2010). Serotype specific invasive capacity and persistent reduction in invasive pneumococcal disease.. Vaccine.

[25] Brueggemann AB, Peto TE, Crook DW, Butler JC, Kristinsson KG (2004). Temporal and geographic stability of the serogroup-specific invasive disease potential of Streptococcus pneumoniae in children.. J Infect Dis.

[26] Lagos R, Munoz A, San Martin O, Maldonado A, Hormazabal JC (2008). Age- and serotype-specific pediatric invasive pneumococcal disease: insights from systematic surveillance in Santiago, Chile, 1994–2007.. J Infect Dis.

[27] Hanage WP, Kaijalainen TH, Syrjanen RK, Auranen K, Leinonen M (2005). Invasiveness of serotypes and clones of Streptococcus pneumoniae among children in Finland.. Infect Immun.

[28] Weinberger DM, Harboe ZB, Sanders EA, Ndiritu M, Klugman KP (2010). Association of serotype with risk of death due to pneumococcal pneumonia: a meta-analysis.. Clin Infect Dis.

[29] Dagan R, Givon-Lavi N, Greenberg D, Fritzell B, Siegrist CA (2010). Nasopharyngeal carriage of Streptococcus pneumoniae shortly before vaccination with a pneumococcal conjugate vaccine causes serotype-specific hyporesponsiveness in early infancy.. J Infect Dis.

[30] Tyrrell GJ, Lovgren M, Chui N, Minion J, Garg S (2009). Serotypes and antimicrobial susceptibilities of invasive Streptococcus pneumoniae pre- and post-seven valent pneumococcal conjugate vaccine introduction in Alberta, Canada, 2000–2006.. Vaccine.

[31] Dagan R, Klugman KP (2008). Impact of conjugate pneumococcal vaccines on antibiotic resistance.. Lancet Infect Dis.

[32] Kaplan SL, Mason EO, Wald ER, Schutze GE, Bradley JS (2004). Decrease of invasive pneumococcal infections in children among 8 children's hospitals in the United States after the introduction of the 7-valent pneumococcal conjugate vaccine.. Pediatrics.

[33] Talbot TR, Poehling KA, Hartert TV, Arbogast PG, Halasa NB (2004). Reduction in high rates of antibiotic-nonsusceptible invasive pneumococcal disease in tennessee after introduction of the pneumococcal conjugate vaccine.. Clin Infect Dis.

[34] Hsu KK, Shea KM, Stevenson AE, Pelton SI (2010). Changing serotypes causing childhood invasive pneumococcal disease: Massachusetts, 2001–2007.. Pediatr Infect Dis J.

[35] Simoes AS, Pereira L, Nunes S, Brito-Avo A, de Lencastre H (2011). Clonal evolution leading to maintenance of antibiotic resistance rates among colonizing Pneumococci in the PCV7 era in Portugal.. J Clin Microbiol.

[36] Stamboulidis K, Chatzaki D, Poulakou G, Ioannidou S, Lebessi E (2011). The impact of the heptavalent pneumococcal conjugate vaccine on the epidemiology of acute otitis media complicated by otorrhea.. Pediatr Infect Dis J.

